# The Story of the Insane from Year to Year

**Published:** 1889-07-13

**Authors:** 


### The Story of the Insane from Year to Year.

Asylum Reports.

### DERBY COUNTY ASYLUM.

There is accommodation is this asylum for 471 beds, and at the close of the year there were 36 vacancies, or 42 if the six beds at the Farm Cottage be included; 159 patients were admitted, 101 were discharged, and 74 died. The recoveries reach the high percentage of 43·91 on the admissions. The death-rate is 16·29, which is 4 per cent. higher than it was in 1887, and the high mortality is attributed to the unfavourable nature of the admissions. The coroner held four inquests. In one the patient, who appeared to be convalescing from melancholia, committed suicide when working on the farm. There is always a certain amount of risk in employing such patients, but the advantages of occupation are so great that some risk is justifiable. Dr. Lindsay discusses the question of pensions to asylum officers. He shows that twenty-three persons connected with the asylum have been pensioned since the opening of the institution, and the committee of visitors passed a resolution in favour of existing officers having an assured right to their pensions. The minor improvements have been very numerous. Covered passages have been made between some of the wards, electrical bells have been fitted up, new machinery has been fitted up in the kitchen, and alterations made at the farm. We are pleased to learn that the dietary has been revised and improved. The rate of maintenance stands at 9s. 4¼d. Dr. Lindsay thinks there is "too great a tendency to claim credit for a low maintenance rate, obtained too often, probably, by sacrificing the patients' comforts. A very low maintenance rate requires careful scrutiny, should be viewed with suspicion, and is not, in my opinion, a thing to be commended or to boast of." This would all depend on how the low rate was obtained, and everything should be taken into account on estimating the rate. The mere numbers make an important difference; 9s. 4d. might be low for 400, and 7s. 6d. might be high for 1,200 beds. The medical superintendent's report is a very long and exhaustive one, while that of the visitors is very short, and it is questionable whether it complies with the 16 and 17 Vic., c. 97, s. lxii., as to "the state and condition of such asylum, and as to its sufficiency for the proper accommodation of the number of lunatics for whom it may be requisite to provide accommodation, and as to the management of such asylum, and the conduct of the officers and servants thereof, and the care of the patients therein."

### DEVON COUNTY ASYLUM.

This asylum was opened in 1845, and at the end of 1848 it contained 351 patients. Now there are 889 patients resident, and nearly £6,500 have recently been spent on additions. Among other things, a large dining-hall was provided, which will seat 400 patients, and the visitors think it "desirable that they should be so assembled at meals where supervision on the part of attendants and medical men is more easily attained." We think the desirableness of assembling patients at meal times is open to the gravest doubt, and it will probably be found at Devon that the 400 patients who use the hall for their meals are just such as least need supervision, and that the infirm and acute cases will still have to take their meals in the wards. During the year, 166 patients were admitted, 88 were discharged, and 51 died. The recoveries stand at 35·62 per cent., and the deaths at 5·8. The latter is very low for a county asylum; but 11 of the deaths were due to phthisis, and one to enteric fever. Dr. Saunders says: "The medical treatment, occupations and amusements of the patients have been much the same as in former years, and with this class of the community, as with others, the adage in medicine holds good, 'Try all things'; yet little that is new proves to be true, and we have, more or less, to stick to old methods modified by modern experiences." The weekly rate is 7s. 6d. The visitors remark that this is lower than any other asylum; but here they are wrong, as Denbigh, Dorset, Lancaster, Monmouth, Northampton, Worcester, and Wilts are all as low a Devon, and some are lower.

### HULL BOROUGH ASYLUM.

Two hundred and eighty-two patients were in residence at the close of the year, showing an increase of 28, as compared with 1887. One hundred cases were admitted, 52 were discharged, and 29 died. The recoveries stand at 33 per cent. for the year, while the average since the opening of the asylum was 37·96. The deaths were 10·58, as compared with 14·10, showing that the mortality in this asylum is above the average, and this high mortality is in great part owing to the number of paralytics and other unfavourable cases which the borough of Hull produces. Of the admissions not fewer than 23 were owing to alcoholic excess. Dr. Merson gives a most interesting summary of the results obtained during the ten years he has so ably filled the post of medical superintendent. During the decade, 824 patients were admitted, 366 were discharged, 234 died, and 224 remain in the asylum. Dr. Merson proceeds: "Looking at the admissions, I find that of the 824 cases 309 were suffering from forms of mental disease, which rendered a cure from the first impossible, so that only 575 had from the first the remotest chance of recovery, and many even of them more from other circumstances very unfavourable. Forty-five per cent. of them, however, recovered, and 12 per cent. more were discharged considerably improved."

### WILTS COUNTY ASYLUM, DEVIZES.

This asylum, like so many others, adds yearly to the number of its inmates. It now contains 672 patients, and every part of it would seem to be full. One hundred and thirty-one cases were admitted during the year just passed, 51 were discharged, and 66 died. The recoveries stand at 33·6 per cent. on the admissions, and the deaths at 9·9. Not fewer than 10 of the deaths were due to phthisis, and it is extremely likely that the crowded state of the asylum had something to do with this rather high mortality. The asylum is an old one, and it would be interesting to know whether the cubic space allowed is according to the old or the new requirements of the Lunacy Commissioners. Of the causes of insanity, we note that heredity tendency accounts for 38, and drink for only 8. The Wiltshire peasants receive such low wages, that it would not be possible for them to go to great excess in that direction; hence they are saved from one temptation. But the high proportion of insanity in the county is probably in part owing to the low wages given necessitating a poor supply of food. In 8 instances, also, we find old age brought on the disease. It is sad to think that a man should live a long life and then die in an asylum. The water supply has been deficient for some time, and the visitors have now entered into a contract with the Devizes Water Company. A hospital for infectious diseases has been built at an estimated expense of £1,885. Nine builders tendered for the work, and the highest tender was £4,677, and the lowest £3,150. The visitors accepted none of these, and called upon the county surveyor to modify his specifications without altering his plans. This he did, and the builders again set to work, with the result that a tender was received at £1,869. A plan of the hospital is given, and although not free from faults it may be considered very creditable for the sum spent on it, and it is certainly better than the majority of asylum hospitals. The medical superintendent's report is, as usual, a model of clearness.

